# Continuous single cell imaging reveals sequential steps of plasmacytoid dendritic cell development from common dendritic cell progenitors

**DOI:** 10.1038/srep37462

**Published:** 2016-11-28

**Authors:** Ezgi Dursun, Max Endele, Andrea Musumeci, Henrik Failmezger, Shu-Hung Wang, Achim Tresch, Timm Schroeder, Anne B. Krug

**Affiliations:** 1 Institute for Immunology, Biomedical Center, Ludwig-Maximilians-University Munich, Großhaderner Str. 9, 82152 Martinsried, Germany; 2 Department of Biosystems Science and Engineering, ETH Zurich, Mattenstrasse 26, 4058 Basel, Switzerland; 3 Max-Planck-Institute for Plant Breeding Research, Carl-von-Linné-Weg 10, 50829 Cologne, Germany; 4 Department of Biology, University of Cologne, Zülpicher Str. 47, 50829 Cologne, Germany

## Abstract

Functionally distinct plasmacytoid and conventional dendritic cells (pDC and cDC) shape innate and adaptive immunity. They are derived from common dendritic cell progenitors (CDPs) in the murine bone marrow, which give rise to CD11c^+^ MHCII^−^ precursors with early commitment to DC subpopulations. In this study, we dissect pDC development from CDP into an ordered sequence of differentiation events by monitoring the expression of CD11c, MHC class II, Siglec H and CCR9 in CDP cultures by continuous single cell imaging and tracking. Analysis of CDP genealogies revealed a stepwise differentiation of CDPs into pDCs in a part of the CDP colonies. This developmental pathway involved an early CD11c^+^ SiglecH^−^ pre-DC stage and a Siglec H^+^ CCR9^low^ precursor stage, which was followed rapidly by upregulation of CCR9 indicating final pDC differentiation. In the majority of the remaining CDP pedigrees however the Siglec H^+^ CCR9^low^ precursor state was maintained for several generations. Thus, although a fraction of CDPs transits through precursor stages rapidly to give rise to a first wave of pDCs, the majority of CDP progeny differentiate more slowly and give rise to longer lived precursor cells which are poised to differentiate on demand.

Clinical and animal studies provide evidence for an important role of plasmacytoid dendritic cells (pDCs) in innate antiviral defense, systemic and tissue-specific autoimmunity^1^,^2^,^3^ and immunopathology during chronic viral infection^4^ involving their capacity to secrete high amounts of type I interferons (IFNs). Furthermore, pDCs were shown to promote immune tolerance preventing neuroinflammation^5^,^6^ and graft versus host disease after allogeneic bone marrow (BM) transplantation^7^,^8^.

PDC and conventional DC subpopulations are derived from the common dendritic cell progenitor (CDP) population in murine and human BM. PDCs develop from CDP in the BM^9^,^10^ and are retained there at a higher frequency than cDCs, which derive from circulating cDC precursors (pre-cDCs)^11^,^12^. Generation of DC subpopulations is not confined to the CD115^+^ CDP population as CD115^−^ DC progenitor cells in murine BM were also shown to give rise to all DC subtypes with a bias towards pDC generation^13^. PDC development is driven by transcription factor E-protein E2-2/Tcf4, which in turn is controlled by inhibitor of DNA binding 2 (Id2)^14^,^15^. Conversely, E2-2 acts in concert with Myeloid translocation gene 16 (Mtg16) and other factors such as Zeb2^16^ to repress Id2, allowing final pDC differentiation^17^. Several pDC subpopulations have been identified in murine BM and spleen^18^,^19^,^20^,^21^,^22^ as well as in human blood^23^,^24^,^25^,^26^,^27^, which are distinct in phenotype and function. It remains to be elucidated whether these subpopulations represent sequential stages of differentiation and maturation or whether they develop independently of each other. We have previously identified a population of Siglec H^+^ CCR9^low^ precursors in murine BM, which resembles pDCs in phenotype and function. In contrast to pDCs, however, those cells have the capacity to generate mature pDCs or cDC subsets in the steady state depending on the environmental cues provided in different tissues^22^,^28^. This population is characterized by expression of CD11c, Siglec H and BST2 and low expression of CCR9, B220 and MHCII. The Siglec H^+^ CCR9^low^ precursors express E2-2 and produce type I IFNs and other cytokines in response to toll-like receptor (TLR) 7 and 9 stimulation similar to CCR9^high^ pDCs, but they are not yet capable of presenting antigens on MHC class II^29^. Other groups have described Siglec H^+^ pre-DCs, which partially express Zbtb46 and give rise to pDCs and cDC subtypes^30^,^31^. This population was shown to be enriched in the BM of Mtg16-deficient mice due to aberrant Id2 induction in these cells blocking pDC development^17^. Recent work suggested that Siglec H^+^ pre-DCs are derived from CDPs and constitute an early pre-DC stage which gives rise to pDCs and pre-cDCs^17^,^31^. It was unclear so far, if the Siglec H^+^ CCR9^low^ population truly is a CDP-derived precursor of pDCs or if it develops in parallel as an immature subset of pDCs. To clearly delineate the ontogeny and cell fate of this pDC-like precursor population and to understand the extent of lineage commitment at the CDP and pre-DC stages, we chose to study the development of individual CDP progeny *in vitro* by single cell imaging and tracking^32^. This approach allowed us to correlate cell division behaviour and acquisition of cell type defining markers in CDP progeny. Time series analysis elucidated the relationship between cell types, thereby refining the model of differentiation events from CDPs to mature DCs. Using this method, we could show that pDCs develop from CDPs sequentially via intermediate stages of early CD11c^+^ SiglecH^−^ pre-DC and SiglecH^+^ CCR9^low^ precursors.

## Results

### Continuous long-term observation of individual dendritic cell progenitors and their progeny

**Figure 1:**
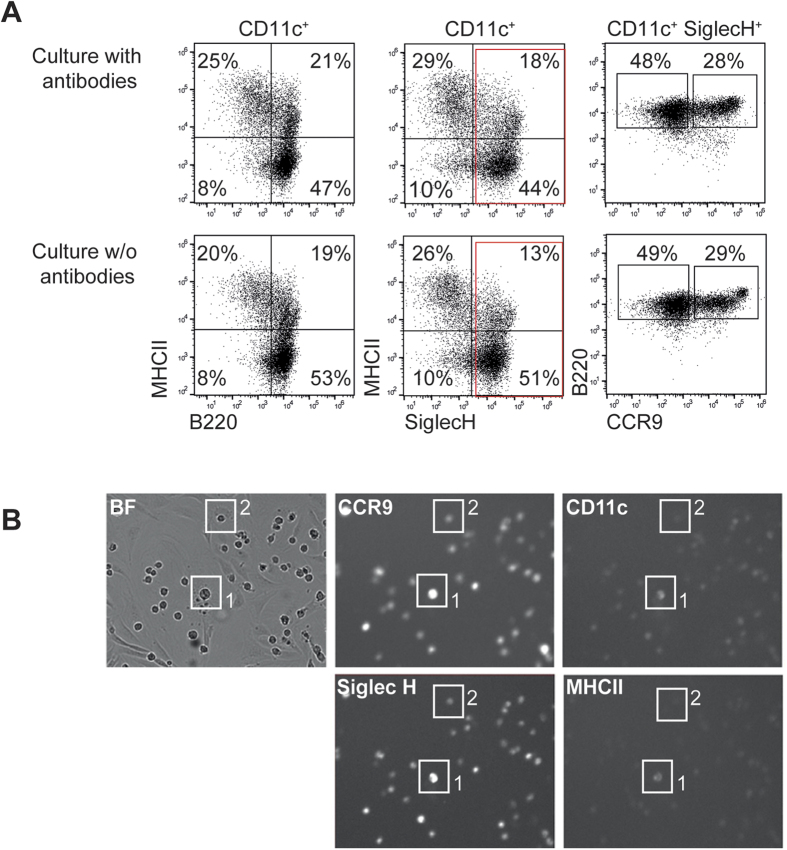
Identification of cell types by in culture staining. (**A**) Total BM cells were cultured either with fluorescently labeled antibodies against SiglecH, CCR9, CD11c and MHCII or without antibodies in the presence of 20 ng/ml Flt3L for 5 days. After 5 days, cells were stained freshly with fluorescently labeled antibodies against SiglecH, CCR9, CD11c, MHCII and B220. The percentages of pDCs, cDCs and SiglecH^+^ CCR9^high^ and SiglecH^+^ CCR9^low^ subpopulations were determined by FACS analysis. Results of one representative of 2 experiments are shown. (**B**) CDPs were cultured on EL08 feeder cells with 20 ng/ml Flt3L for 5 days. Fluorescently labeled antibodies against Siglec H, CCR9, CD11c and MHCII were added at the start of the experiment. Exemplary phase contrast and fluorescence images of indicated cells are shown.

Common DC progenitors (CDP) isolated from murine BM cells give rise to DC subpopulations including pDC and cDCs in culture with Flt3L and feeder cells or *in vivo* after adoptive transfer. Recent studies indicate that CDP give rise to CD11c^+^ MHCII^−^ pre-DC populations, which are biased to differentiate into specific DC subpopulations. The precise steps of pDC and cDC development from the CDP and the relationship between individual progenitor, precursor and differentiated cells are not known. To reveal the genealogy of the DC lineage and identify sequential steps of pDC development, CDP and their progeny were continuously imaged and tracked. To reduce the heterogeneity of the CDP population CD115^−^ DC progenitor cells^13^ were excluded (Supplementary Fig. 1). A co-culture system was established which allows distinction of CDP and their progeny from the feeder cell layer by morphology. The embryonic liver (EL) 08 stromal cell line supported CDP expansion and differentiation into pDCs and cDCs in the presence of Flt3L similar to total BM feeder cells (Supplementary Fig. 2). For continuous imaging, sorted CDP were cultured on EL08 monolayers with Flt3L in μ-slides for 5 days. Acquisition of CD11c, MHC II, Siglec H and CCR9 expression was detected by continuous in culture staining^33^. Addition of diluted fluorescently labeled antibodies during FL-DC cultures did not affect the phenotype and frequency of DC subsets, which were generated (Fig. 1A). The sensitivity of the fluorescence imaging was sufficient to detect all markers and to distinguish CCR9^high^ pDCs (e.g. cell no. 1) from SiglecH^+^ CCR9^low^ cells (e.g. cell no. 2) (Fig. 1B). CDP pedigrees with annotation of the fluorescent markers (off, on or high) were generated by single cell tracking (Supplementary Fig. 3) from different imaging positions (exp. 1: 40 annotated pedigrees, containing 676 cells; exp. 2: 45 pedigrees containing 1371 cells). Apoptosis occurred in only 3.7% and 0.9% of the cells and 17.2% and 16.6% of the CDP progeny were lost to tracking due to movement outside of the imaging position or migration under the stromal cell layer (data from exp. 1 and exp. 2, respectively, data not shown). Within the remaining non-apoptotic and not-lost cells, over 50% were dividing during the experiment time (exp. 1: 56.8%, exp. 2: 58%, data not shown) and up to 8 generations could be observed. Thus, continuous imaging of CDP in the EL08/Flt3L co-culture system allows the generation of CDP genealogies, which reveal heterogeneous behaviour of CDP progeny with regard to number of divisions and acquisition of cell type defining markers.

### Sequential development of pDCs via distinct precursor stages

**Figure 2:**
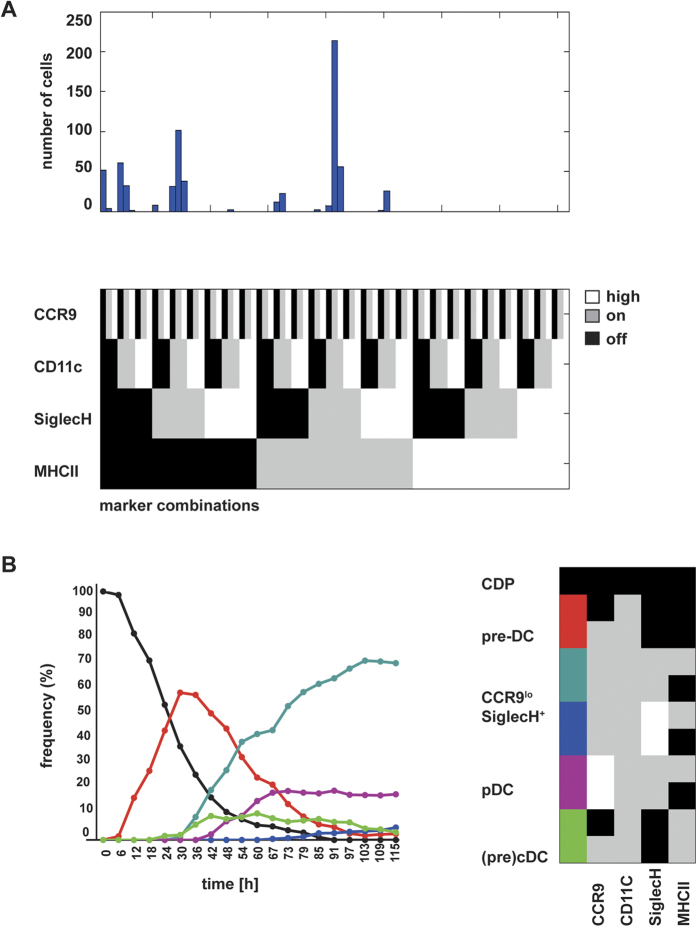
Sequential appearance of cell types in CDP cultures. (**A**) Frequency of marker combinations. Each marker can have 3 states, according to its expression level: off (black), on (grey) or high (white). Each cell is counted once, with its marker combination observed immediately before cell division. (**B**) Marker combinations were grouped manually into distinct cell types (right panel). The relative frequency of each cell type is shown for each time point.

The frequency of all possible marker combinations was determined for all cells within the pedigrees occurring during the 120 hours of experiment time. SiglecH^+^ CCR9^low^ precursor cells with the annotation CCR9 on, CD11c on, Siglec H on, MHCII on or off were the most frequent cell type observed (51.2%, Fig. 2A and B). These cells contained a small percentage of cells with higher intensity of the Siglec H signal (4.2% of SiglecH^+^ CCR9^low^), which was due to intracellular accumulation of the Siglec H antibody or upregulation of Siglec H expression. The second most frequent cell types were early pre-DC (CD11c on, CCR9 on or off, Siglec H off, MHCII off, 13.9%) and CCR9^high^ pDCs (CCR9 high, CD11c on, Siglec H on or high, MHCII on or off, 14%). CDP (all markers off, 7.8%) and cells with cDC or pre-cDC phenotype (CCR9 on or off, CD11c on, Siglec H off, MHCII on, 5.1%) were detected at lower frequencies. A clear distinction between pre-cDCs and cDCs was not possible because MHCII can already be expressed at low levels in pre-cDCs. While the CDP population was rapidly declining, the pre-DC population increased with a peak at approximately 30 hours (Fig. 2B). The number of pre-DCs decreased thereafter concomitant with an increase in the SiglecH^+^ CCR9^low^ precursor population, which was followed immediately by an expansion of the pDC population. cDCs/pre-cDCs appeared quite early together with the SiglecH^+^ CCR9^low^ precursors and the population was maintained thereafter (Fig. 2B). The sequential appearance of the different populations indicates progressive development of the CDP progeny via distinct precursor stages.

**Figure 3:**
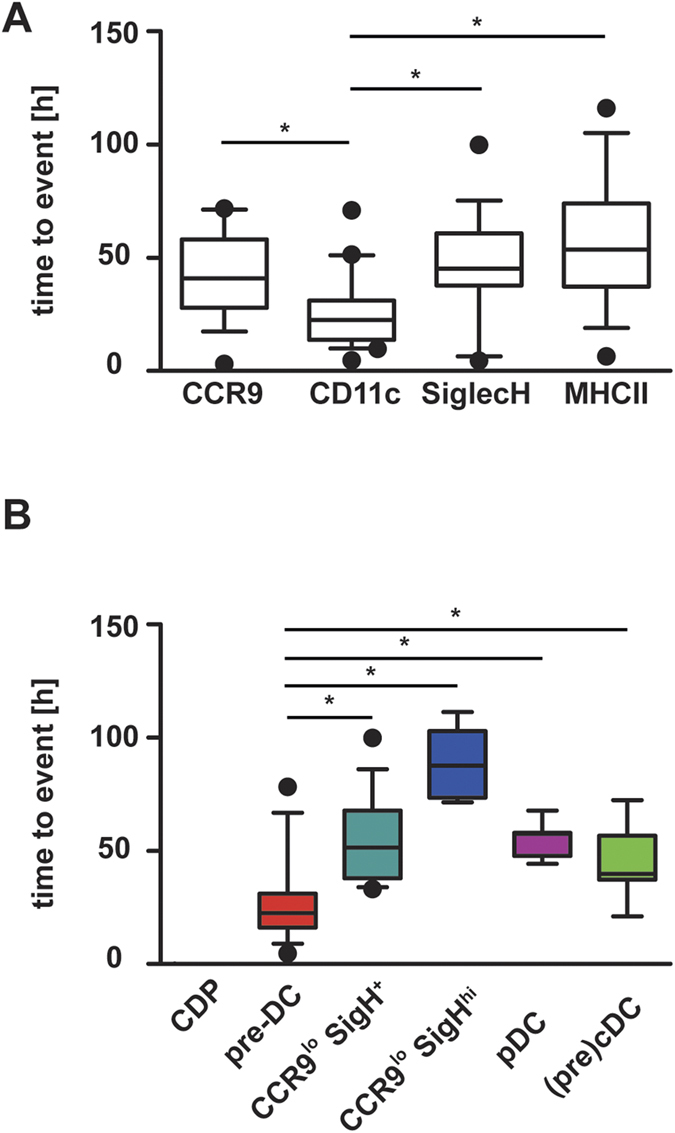
Time-dependent occurrence of cell types in CDP genealogies. CDPs were cultured on EL08 feeder cells with Flt3L for 5 days and their progeny were observed continuously by single cell imaging and tracking. (**A**) The time until first occurrence of fluorescent markers in the pedigrees was determined. (**B**) The time until first occurrence of each cell type in the pedigrees was calculated as weighted mean. Data are shown as box plots with whiskers and outliers, median values indicated by horizontal lines. Significant differences are indicated by an asterisk (p < 0.05, Kruskall-Wallis-test with Dunn’s post test).

Analysis of the time of onset of the marker expression in the pedigrees showed that CD11c was the earliest marker to be expressed (median 22.5 hrs), followed by CCR9 (40.9 hrs), Siglec H (45.1 hrs) and then MHC class II (53.6 hrs) (Fig. 3A). The onset of high signal intensity of CCR9 and Siglec H occurred later in the pedigrees (median 54.3 and 71.3 hrs respectively) (data not shown). The sequence of time of onset of CD11c, CCR9 and SiglecH was confirmed in a second experiment (Supplementary Fig. 4A,B). The time until occurrence of the different cell populations defined by marker combinations was calculated as the weighted average of the individual waiting times within each pedigree. The median time to event was shortest for pre-DCs (22 hrs) followed by SiglecH^+^ CCR9^low^ precursors (51 hrs) and then pDCs (58 hrs). The first appearance of (pre)-cDCs (CD11c^+^ MHCII^+^ Siglec H^−^ cells) was observed later than that of pre-DCs, but earlier than that of pDCs (40 hrs). The small subset of SiglecH^high^ CCR9^low^ cells appeared at later time points (88 hrs) (Fig. 3B). From these results we conclude, that on a population basis CD11c^+^ MHCII^−^ Siglec H^−^ early pre-DCs precede SiglecH^+^ CCR9^low^ cells which are followed by CCR9^high^ pDCs shortly thereafter. Cell phenotypes were inherited by the daughter cells in most cases, but at later time points acquisition of additional markers by the daughter cells was not synchronized and quite heterogeneous.

**Figure 4:**
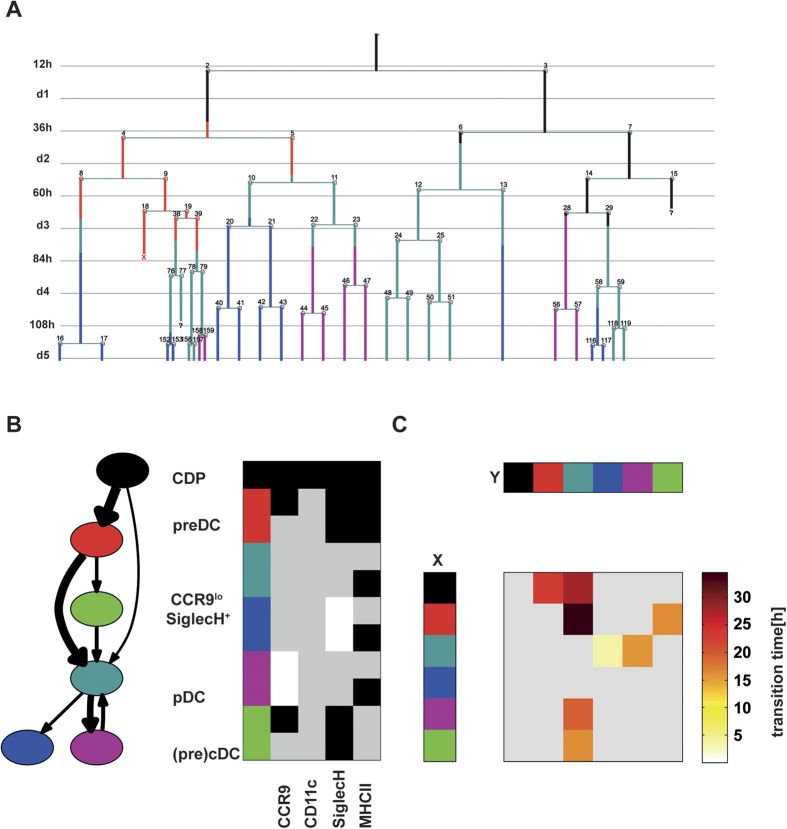
Frequency and duration of transitions between cell types. (**A**) A representative pedigree of a single CDP and its progeny. Branches are colored according to cell type (see Fig. 4B). Apoptotic events are marked with X whereas cell losses are indicated by a question mark. (**B**) Cell type transition graph. Nodes represent cell types defined by marker combinations. The colour code and assigned marker combinations are shown for each cell type on the right. The number of observed transitions between two cell types defines the width of the connecting arrow. Transitions occurring less than 10 times were omitted. (**C**) Median waiting time spent by cell type x (rows) until transformation into cell type y (columns). Infrequent transitions (observed less than 10 times) and the main diagonal are greyed out.

Direct transition between cell types was analysed in the pedigrees (see example shown in Fig. 4A). The number of transitions between cell types in all pedigrees were counted (Fig. 4B, Supplementary Table 1). Transitions between CDP and pre-DC and between pre-DC and SiglecH^+^ CCR9^low^ cells were observed frequently. Simultaneous acquisition of CD11c, CCR9 and SiglecH signals (SiglecH^+^ CCR9^low^ cell phenotype) was observed (e.g. Fig. 4A. cell no. 6 and 29), but the most frequent transition to SiglecH^+^ CCR9^low^ cells was from pre-DCs (CD11c^+^ CCR9^−^ or CCR9^+^ phenotype). In the majority of the cases CCR9^high^ pDCs developed from SiglecH^+^ CCR9^low^ cells (exp. 1: 87%, Fig. 4B; exp. 2: 65%). Direct acquisition of the pDC phenotype by CDP or pre-DC was also observed (e.g. Fig. 4A, cell no. 28). Although the high CCR9 signal was transmitted to the daughter cells (e.g. Fig. 4A cells no. 22, 23, 28), it was not always maintained at high levels in pDCs until the end of the experiment. Calculation of the transition times between cell types (Fig. 4C) revealed that transition from SiglecH^+^ CCR9^low^ precursors to CCR9^high^ pDCs was rapid (median/IQR: 16/15 hrs) compared to the transition from CDP and pre-DC to SiglecH^+^ CCR9^low^ precursors (median/IQR: 28/39hrs and 35/32 hrs respectively). Taken together our results show that a part of the CDPs were able to give rise to pDCs within 3 days of culture and in the majority of these cases CDP progeny transited sequentially through pre-DC and SiglecH^+^ CCR9^low^ intermediate precursor stages of variable duration before differentiating into pDCs.

### Heterogeneous behaviour of CDP progeny regarding number of generations and cell fate

**Figure 5:**
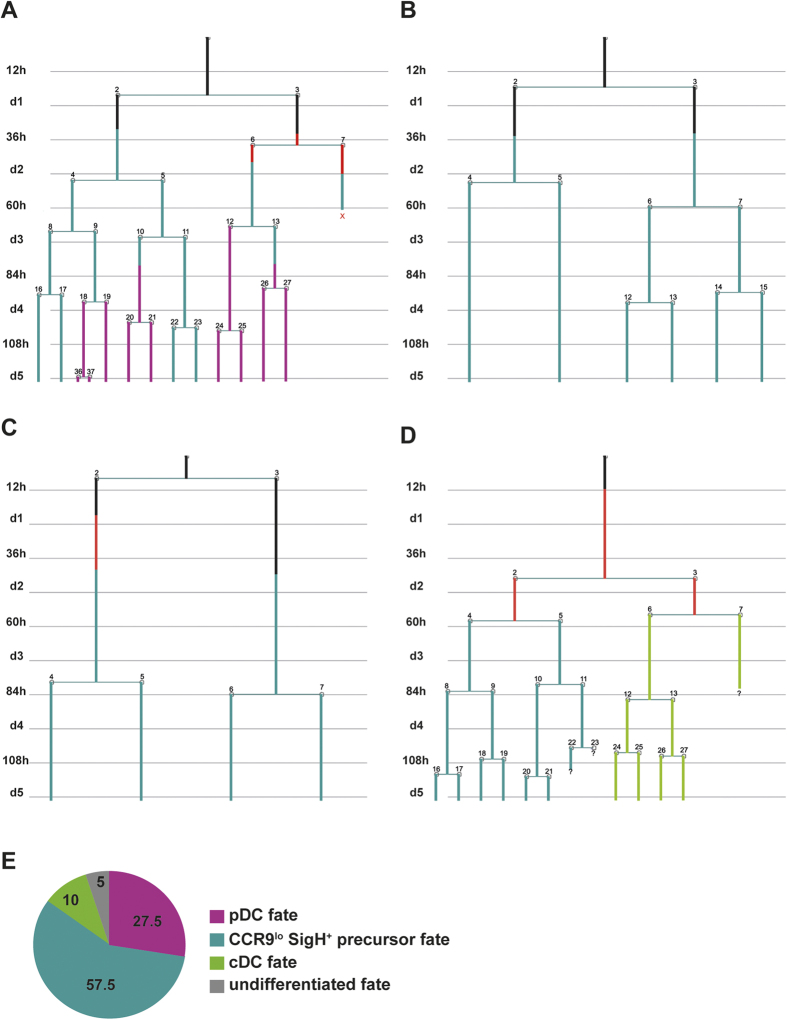
Heterogeneity of CDP division kinetic and cell fate. CDP and their progeny were monitored by time-lapse microscopy and single cell tracking. Cell divisions and fluorescent signals were annotated in pedigrees. Pedigrees were segregated according to their cell fate. Examples of pedigrees with pDC and SiglecH^+^ CCR9^low^ fate (**A**), with only SiglecH^+^ CCR9^low^ fate (**B**,**C**) or with SiglecH^+^ CCR9^low^ and cDC fate (**D**) are shown. (**E**) The pie chart shows the percentages of pedigrees, in which generation of pDCs or cDCs occurred, in which SiglecH^+^ CCR9^low^ precursor cells were generated without occurrence of pDCs or cDCs, and, in which only cells with CDP or pre-DC phenotype were generated (undifferentiated).

Evaluation of the pedigrees revealed that CDP were heterogeneous in the number of generations and colony size they produced. After exclusion of nine pedigrees, in which many of the cells could not be observed until the end of the experiment, the median number of generations in the pedigrees was 4 (IQR 2) and the median colony size was 19 cells (IQR 16, n = 31). From the third generation onwards, sibling cells and their progeny showed highly heterogeneous behaviour regarding cell life time and number of divisions (see Figs 4A and 5A–D).

CDP and their progeny were also heterogeneous with regard to the cell fate they reached during the experiment time (Fig. 5E, Supplementary Fig. 4). In approximately one third of the pedigrees (exp. 1: 27.5%, exp. 2: 36. %) a pDC cell fate was observed, including pedigrees in which both pDC and CCR9^low^ precursor cells were found at the end of the experiments (examples shown in Figs 4A and 5A). In a considerable number of pedigrees CDP generated Siglec H^+^ CCR9^low^ precursors, but not CCR9^high^ pDCs (exp.1: 57.5%, exp. 2: 33%; examples shown in Fig. 5B and C). In these cases the Siglec H^+^ CCR9^low^ precursor state was maintained until the end of the observation time. In a minority of the pedigrees, CD11c^+^ Siglec H^−^ MHCII^+^ (pre)-cDC were observed as final cell phenotype including pedigrees with additional generation of SiglecH^+^ CCR9^low^ precursors (exp. 1: 10%, exp. 2: 9%; example shown in Fig. 5D). The percentage of pedigrees, which maintained an undifferentiated phenotype (expressing only CD11c and/or CCR9) varied between experiments (exp.: 5%, exp. 2: 22%). Generation of cDC/pre-cDC and CCR9^high^ pDCs from the same CDP was not observed during the experiment time, suggesting that CDP were already precommitted to the pDC or cDC lineage. Taken together, cell fate analysis in CDP genealogies showed that rapid differentiation into pDCs occurred in a part of the colonies, whereas the CD11c^+^ SiglecH^+^ CCR9^low^ phenotype was maintained for several generations in the majority of the remaining colonies. To investigate the differentiation potential of SiglecH^+^ CCR9^low^ precursors in our culture conditions SiglecH^+^ CCR9^low^ cells and CCR9^high^ pDCs were sorted from primary BM cells and their phenotype was analyzed by flow cytometry after 48 hrs of coculture with EL08 cells in the presence of Flt3L or Flt3L and GM-CSF (see Supplementary Fig. 5). The majority of SiglecH^+^ CCR9^low^ precursors upregulated CCR9 confirming that they can differentiate into pDCs under steady state conditions. Addition of GM-CSF to the culture prevented pDC differentiation in a part of these cells, while the other part of this population was committed to generate CCR9^high^ pDCs. However the phenotype of CCR9^high^ pDCs (sorted from primary BM cells) was not changed under these conditions (Supplementary Fig. 5).

**Figure 6:**
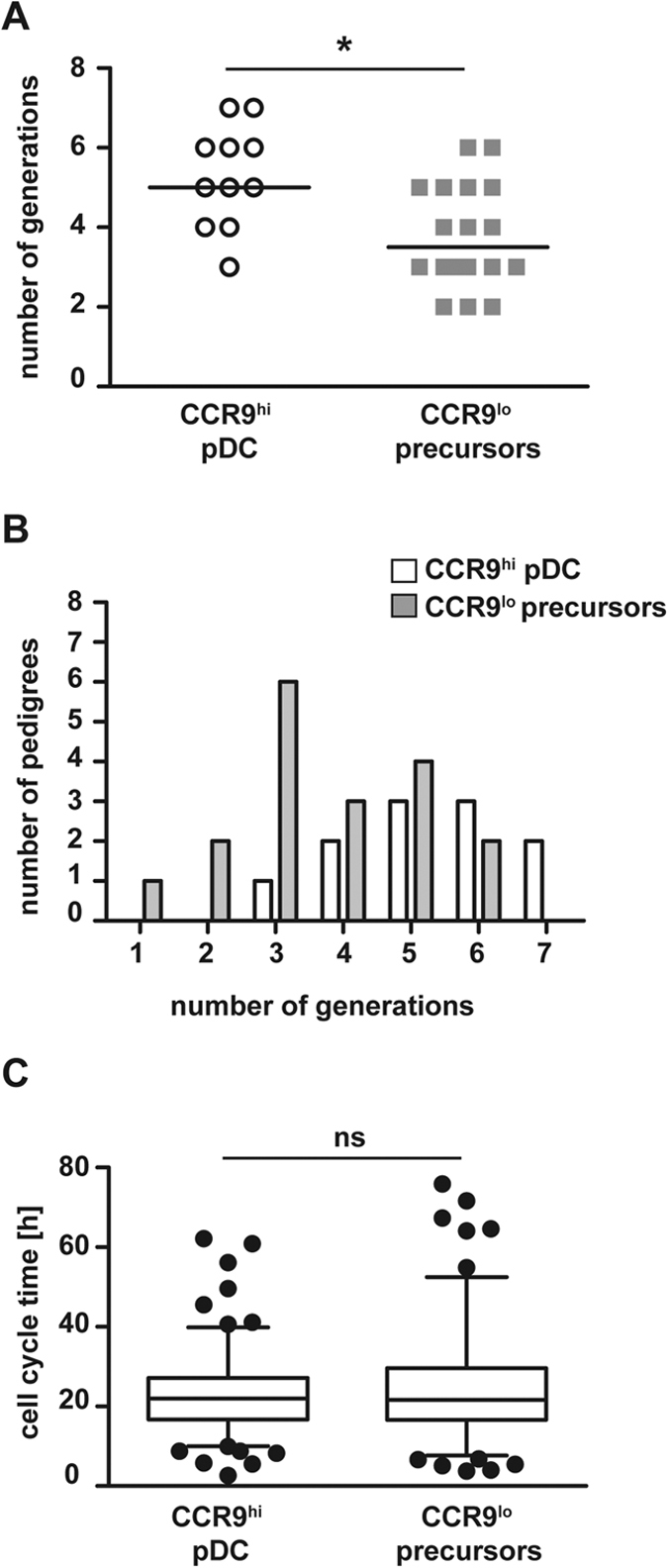
Number of divisions correlates with differentiation stage. (**A**) CDPs were cultured on EL08 stromal cells for 5 days and progenies of the CDP were tracked continuously. Pedigrees were segregated according to their cell fates. The numbers of generations in each pedigree are shown as symbols (CCR9^high^ pDC fate, n = 11 vs. only Siglec H^+^ CCR9^low^ precursor fate, n = 18). Median numbers of generations are indicated by lines. *p = 0.0055, unpaired t-test. (**B**) The numbers of pedigrees with the indicated number of generations are shown for pedigrees with CCR9^high^ pDC fate (white columns) and only Siglec H^+^ CCR9^low^ precursor fate (grey columns). (**C**) The cell life times of all dividing cells in pedigrees with CCR9^high^ pDC fate (n = 153 cells) and Siglec H^+^ CCR9^low^ precursor fate (n = 134 cells) are shown (ns: not significant, unpaired t-test).

A higher number of generations was observed in pedigrees, which generated pDCs during the experiment time, compared to pedigrees which generated only Siglec H^+^ CCR9^low^ cells (median 5 vs. 3.5, p = 0.0055, Fig. 6A and B). A similar trend towards a higher number of generations in CDP colonies with pDC fate was observed in a second independent experiment (Supplementary Fig. 4). In all pedigrees with pDC development, pDCs continued to divide. The median cell lifetime of dividing cells was similar for pedigrees with pDC generation and for pedigrees, which generated only CCR9^low^ precursors (22.0 vs. 21.5 hrs, Fig. 6C). Our results suggest that CDP differ with regard to speed of differentiation. Thereby a pool of precursor cells, which is not yet fully committed, can be generated alongside the differentiated cells providing flexibility to the system.

## Discussion

In this study we used for the first time the method of continuous single cell imaging and tracking to observe the sequential development of DCs from individual progenitor cells over time. This method has been previously used to investigate the differentiation of embryonic and adult stem cells^34^,^35^, hematopoietic progenitors^32^,^36^ and cancer cells^37^. Using the CDP/EL08/Flt3L culture system, DC development could be observed continuously at the single cell level for 5 days without disturbing the culture. The in-culture staining technique was used to follow the onset of expression of cell type defining surface markers without influencing the phenotype and frequency of the cell types generated in the culture system. This method has been used previously to track the expression of surface markers on hematopoietic cells developing from haemogenic endothelial cells^38^. It allows the detection of onset of marker expression, whereas the detection of downregulation of surface marker expression may be confounded by antibody internalization. Signals due to intracellular accumulation of antibodies would be expected to decrease by distribution to the daughter cells. However, signal intensity was not reduced after division demonstrating accurate detection of surface marker expression by this method. Furthermore, cells, which were labeled positively for Siglec H and showed intracellular signal, did not lose Siglec H surface expression during the experiment time. Thus, in culture staining accurately detects surface marker combinations, which define cell types developing in CDP cultures. In this study we followed the transitions between different precursor and differentiated cell types that were defined *a priori* by the expression pattern of four different cell surface markers. The *de novo* definition of cell types from time lapse images has recently attracted some interest^39^,^40^. However, the numerous cell divisions in our data create large trees, which require extending the commonly used hidden Markov models to tree-shaped structures (Failmezger *et al*., submitted manuscript). This will yield an unsupervised classification of cells according to surface marker combinations and other phenotypic features.

Our single cell tracking results showed that CDPs gave rise to early CD11c^+^ SiglecH^−^ pre-DCs and then developed into Siglec H^+^ CCR9^low^ precursors. In a subset of CDP colonies, Siglec H^+^ CCR9^low^ precursors rapidly gave rise to CCR9^high^ pDCs, which continued to expand. At the same time, CDP colonies with delayed differentiation produced Siglec H^+^ CCR9^low^ precursors, which maintained this phenotype until the end of the experiments. These cells constitute a sizable population in the BM and contain cells which are poised to differentiate into pDCs as shown previously^22^,^28^. This may be beneficial in the setting of viral infection when pDCs are depleted and need to be replenished rapidly^41^,^42^. Thus, pDC frequency is regulated at the level of proliferation of pDCs in the BM and at the level of differentiation of SiglecH^+^ CCR9^low^ precursors. *In vivo* pDC numbers are additionally regulated by pDC release from BM into the blood and recruitment to lymphoid and non-lymphoid organs, which are controlled by expression of transcription factor Runx2 in pDCs^43^,^44^. CDC numbers were shown to be regulated by release and recruitment of pre-cDCs and local expansion^45^.

Our finding, that Siglec H^+^ CCR9^low^ precursors were generated from SiglecH^−^ pre-DCs is seemingly contradictory to published results, which suggest that Siglec H^−^ pre-DCs are derived from Siglec H^+^ pre^−^DCs and have lost pDC potential^30^,^31^. The SiglecH^−^ CD11c^+^ MHCII^−^ precursors, which we have observed as an intermediary cell type, occurring at early time points in CDP cultures, are not identical to the SiglecH^−^ pre-DC populations found in the primary BM^31^. In our experiments the onset of CD11c expression clearly preceded the onset of Siglec H expression and we found no evidence for downregulation of Siglec H at later time-points, although Siglec H antibody internalization may have masked downregulation of Siglec H expression in our study.

Colonies in which pDCs were generated contained on average more generations, suggesting that division speed and differentiation are connected. But CDPs and even siblings within the same CDP colony were quite heterogeneous regarding division kinetic and cell fate. PDC differentiation was seen alongside SiglecH^+^ CCR9^low^ precursor development in the same pedigrees, showing that CDP colonies as a whole, but also branches within the same CDP colony can differentiate at a different pace and give rise to different developmental stages at the same time point. Even though we excluded the recently described pDC-biased CD115^−^ DC progenitor cells^13^ and restricted our analysis to CD115^+^ CDPs, which show quite homogeneous surface marker expression, single cell tracking revealed the diversity of this population. The heterogeneous behaviour of CDPs and pre-DCs observed in our study is in line with results of clonal assays where only a minority of CDPs gave rise to all DC subtypes^12^,^13^ and with single cell transcriptome data showing great heterogeneity on the gene expression level in DC progenitor cells^31^. The single cell imaging and tracking approach allowed us to identify CDP subpopulations with defined characteristics. Similarly, clusters of CDPs and pre-DCs with common gene expression signatures indicating DC subtype priming were identified by single cell transcriptome analysis^31^. Distinct behaviour and cell fate of progenitor and precursor subsets are either intrinsically predetermined or caused by differential responses to extrinsic factors, such as growth factors and cell contact dependent signals. In the present study, contact with stromal cells and with developing DCs may have influenced the behaviour of individual CDP and their progeny. The situation *in vivo* is similar as hematopoiesis including DC development occurs in BM niches. The culture system used here mimics this situation, but cannot recapitulate the complexity of DC development in the BM *in vivo*. Nevertheless, the heterogeneous behaviour of DC progenitors found in our study was also seen *in vivo* in a study that followed the cell fates of individual progenitor cells marked with heritable DNA barcodes^46^. Our results are in line with the model of early lineage branching in myelopoeisis that is supported by recent publications employing single cell transcriptome analysis and single cell lineage tracing^47^,^48^.

This is the first study, which continuously follows individual DC progenitor cells during their development into functionally distinct DC subpopulations. Our results show that the behaviour of committed DC progenitor cells with regard to division kinetics and acquisition of cell type defining markers is more heterogeneous than previously thought, allowing for plasticity until late steps of development.

## Methods

### Mice

C57BL/6 and CD45.1-congenic mice were bred in our facility under specific pathogen free conditions. All experimental procedures involving mice were performed in accordance with the regulations of and were approved by the local government (Regierung von Oberbayern).

### Cell isolation and culture

BM cells were isolated from femora and tibiae of 6–8 weeks old mice. After red blood cell lysis (RBC Lysis Buffer, Sigma-Aldrich, St. Louis, MO, USA) cells were washed and resuspended in dendritic dell (DC) medium containing RPMI 1640, (Biochrom, Berlin, Germany), 10% (v/v) heat-inactivated fetal calf serum (PAA, Austria), 1% (v/v) non-essential amino acids (PAA), 1% (v/v) Glutamax (Thermo Fisher Scientific, Waltham, MA, USA), 1% (v/v) Sodium Pyruvate (100 mM, Thermo Fisher Scientific), 1% (v/v) penicillin/streptomycin (PAA), 0.05 mM β-mercaptoethanol (Sigma-Aldrich, Germany) and supplemented with 20 ng/mL Flt3L (purified from supernatant of CHO-flk2 cell line kindly provided by Nicos Nicola, Walter and Eliza Hall Institute). Cells were cultured for 5 days at 37 °C in 5% CO_2_ to obtain FL-DCs.

Live cell imaging experiments were performed using the embryonic liver (EL) 08 stromal cell line as feeder cells^49^. EL08 cells were cultured in EL08 medium (MEM-α-Glutamax (Invitrogen, Karlsruhe, Germany), 15% v/v heat inactivated fetal calf serum, 5% v/v horse serum (Stem Cell Technologies, Köln, Germany), 1% penicillin/streptomycin, 0.01 mM β-mercaptoethanol) at a density of 5*10^5^/ml in 10 cm dishes coated with 0.1% gelatin (Sigma Aldrich, Seelze, Germany) as described before^49^. After 2–3 days of expansion, cells were detached using Trypsin/EDTA (Thermo Fisher Scientific), resuspended in EL08 medium and seeded in ibidi μ-slides (Ibidi, Martinsried, Germany) for imaging experiments.

### Flow Cytometry and cell sorting

For isolation of CDPs, lineage-positive cells were depleted from BM cells by staining with FITC-conjugated antibodies against CD3, CD19, B220, Gr1, NK1.1 and CD11b in FACS buffer (PBS containing 2% FCS) and separation using anti-FITC microbeads (Miltenyi Biotech, Germany). Lineage-depleted cells were stained with FITC-conjugated antibodies against CD3, CD19, B220, Gr-1, NK1.1 (BD Biosciences), CD11b (eBioscience) and with anti-CD135-PE, anti-CD11c-PE-Cy7, anti-CD115-APC, anti-CD117-eFlour780 and anti-MHCII-eFlour450 (eBioscience) in FACS buffer (PBS, 2% FCS) containing 50% supernatant of 2.4G2 hybrioma (Fc block) for 20 min on ice. To exclude dead cells from the analysis, propidium ioidide (2.5 μg/ml) was added to each sample just prior to FACS analysis. CDPs were sorted as Lin^−^ Flt3^+^ CD117^low^ CD115^+^ CD11c^−^ MHCII^−^ cells to high purity using FACS Aria III (BD Biosciences), as shown in supplementary Fig. 1. FACS analysis of DC cultures was performed using anti-CD45.1-Pe-Cy5.5, anti-CCR9-PE, anti-CD11c-Pe-Cy7, anti-MHCII-eFluor780 (eBioscience), anti-CD45.2-eFluor450, anti-B220-BV605 (BD Biosciences) anti-SiglecH-A488 and anti-SiglecH-A647 (produced in our lab from 440c hybridoma, kindly provided by Marco Colonna, Washington University, St. Louis, USA). Samples were acquired on a FACS Gallios or Cytoflex flow cytometer (Beckman Coulter, Krefeld, Germany) and analysed using FlowJo software (Tree Star, Stanford, USA).

### Live cell imaging and long-term antibody staining of CDP cultures

Prior to imaging, μ-slides (I^0.4^ Luer Series, uncoated, Ibidi) were coated with 0.1% gelatin and EL08 stromal cells (2*10^4^ cells in 100 μl EL08 medium) were seeded. Within 24 hours, when stromal cells had reached 40–50% optical confluence, the medium was removed and 2*10^3^ CDPs per slide were seeded in DC medium containing 20 ng/ml Flt3L for live cell imaging experiments. Higher concentrations of Flt3L had no major effect on DC subtype differentiation but increased cell density (data not shown) and were therefore less suitable for single cell tracking. Fluorescently labeled antibodies were added for in culture staining as described before^38^. Antibodies were used at the lowest concentrations which provided sufficient fluorescent signals (0.05 μg/ml anti-MHCII-eFluor450, 0.025 μg/ml anti-CD11c-A488, 0.25 μg/ml anti-SiglecH-A647 0.05 μg/ml anti-CCR9-PE). During imaging, culture medium was not replenished and no additional antibodies or Flt3L were added. Time-lapse imaging was performed with a cell observer system (Zeiss, Oberkochen, Germany) at constant 37 °C and 5% CO_2._ Bright field images were taken at 2 min intervals while fluorescent images were taken at 3–4 h intervals with an Axiocam-HRm camera (1338 × 1040 pixel resolution) with 10X objective. Carl Zeiss AxioVision 4.5 Software was used in this study.

### Single cell tracking and analysis of cell fates in pedigrees

Single cell tracking was performed using tracking software (TTT^50^). The software allows loading of acquired phase contrast and fluorescence images with determined intervals. Individual cells were tracked and properties such as lost events, cell death, division, and fluorescent signals were logged manually by the researchers. All relevant information saved during tracking was annotated in pedigrees including fluorescent markers (off, on or high). The raw data consisted of 72 trees originating from single CDP cells. In 32 out of 72 cases (44%), CDP colonies were lost early in the CDP or pre-DC stage, because they could not be tracked further or underwent apoptosis. These colonies were excluded from further analysis. Cell fates were assigned to CDP colonies by visual analysis of annotated pedigrees (results shown in Fig. 5E). Cell types were identified by predefined marker combinations. If a pDC or cDC phenotype was observed in the pedigree, it was assigned a pDC or cDC cell fate. If a SiglecH^+^ CCR9^low^ phenotype was observed in a pedigree without upregulation of CCR9 it was assigned a SiglecH^+^ CCR9^low^ phenotype. If no marker or only CD11c or CCR9 were expressed in a pedigree it was assigned an undifferentiated cell fate.

### Statistical analysis

Single cell imaging data was further analysed using self-written statistical software (D-BSSE, Basel, Switzerland) and Matlab. Two-group comparisons of normally distributed variables were performed by an unpaired two-sided t-test. Alternatively, data which did not follow a normal distribution or showed unequal group variances, was analysed using the non-parametric Kruskal-Wallis test followed by Dunn’s multiple comparison test (GraphPad Prism). P values below 0.05 were considered statistically significant. Data are shown as box and whisker plots indicating median, interquartile range and outliers. Time to event was calculated as a weighted mean 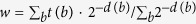, where the sum is taken over cells b at which the event occurs for the first time, t(b) is the time until the event occurred in cell b, and d(b) is the depth at which the event occurred, i.e., the number of cell divisions preceding the event in the respective branch. Transitions between cell phenotypes, were counted at all time points and depicted in a transition graph where the width of the connecting arrows indicates the number of transitions. The transition time between cell types was calculated as the time between the respective events (marker combinations) in the genealogy tree. The whole tree was taken into account. If a marker combination was kept over several generations, the transition time for every single cell was calculated.

## Additional Information

**How to cite this article**: Dursun, E. *et al*. Continuous single cell imaging reveals sequential steps of plasmacytoid dendritic cell development from common dendritic cell progenitors. *Sci. Rep.* **6**, 37462; doi: 10.1038/srep37462 (2016).

**Publisher’s note:** Springer Nature remains neutral with regard to jurisdictional claims in published maps and institutional affiliations.

## Supplementary Material

Supplementary Information

## References

[b1] Rowland S. L. . Early, transient depletion of plasmacytoid dendritic cells ameliorates autoimmunity in a lupus model. J Exp Med 211, 1977–1991 (2014). 25180065 10.1084/jem.20132620 PMC4172228

[b2] Sisirak V. . Genetic evidence for the role of plasmacytoid dendritic cells in systemic lupus erythematosus. J Exp Med 211, 1969–1976 (2014). 25180061 10.1084/jem.20132522 PMC4172218

[b3] Swiecki M. & Colonna M. The multifaceted biology of plasmacytoid dendritic cells. Nat Rev Immunol 15, 471–485 (2015). 26160613 10.1038/nri3865 PMC4808588

[b4] Li H., Evans T. I., Gillis J., Connole M. & Reeves R. K. Bone marrow-imprinted gut-homing of plasmacytoid dendritic cells (pDCs) in acute simian immunodeficiency virus infection results in massive accumulation of hyperfunctional CD4+ pDCs in the mucosae. J Infect Dis 211, 1717–1725 (2015). 25489000 10.1093/infdis/jiu671

[b5] Irla M. . MHC class II-restricted antigen presentation by plasmacytoid dendritic cells inhibits T cell-mediated autoimmunity. J Exp Med 207, 1891–1905 (2010). 20696698 10.1084/jem.20092627 PMC2931160

[b6] Loschko J. . Antigen Targeting to Plasmacytoid Dendritic Cells via Siglec-H Inhibits Th Cell-Dependent Autoimmunity. J Immunol 187, 6346–6356 (2011). 22079988 10.4049/jimmunol.1102307

[b7] Auletta J. J., Devine S. M. & Waller E. K. Plasmacytoid dendritic cells in allogeneic hematopoietic cell transplantation: benefit or burden? Bone Marrow Transplant 51, 333–343 (2016). 26642333 10.1038/bmt.2015.301 PMC4862020

[b8] Hadeiba H. . CCR9 expression defines tolerogenic plasmacytoid dendritic cells able to suppress acute graft-versus-host disease. Nat Immunol 9, 1253–1260 (2008). 18836452 10.1038/ni.1658 PMC2901237

[b9] Naik S. H. . Development of plasmacytoid and conventional dendritic cell subtypes from single precursor cells derived *in vitro* and *in vivo*. Nat Immunol 8, 1217–1226 (2007). 17922015 10.1038/ni1522

[b10] Onai N. . Identification of clonogenic common Flt3+M-CSFR+ plasmacytoid and conventional dendritic cell progenitors in mouse bone marrow. Nat Immunol 8, 1207–1216 (2007). 17922016 10.1038/ni1518

[b11] Liu K. . *In vivo* analysis of dendritic cell development and homeostasis. Science 324, 392–397 (2009). 19286519 10.1126/science.1170540 PMC2803315

[b12] Naik S. H. . Intrasplenic steady-state dendritic cell precursors that are distinct from monocytes. Nat Immunol 7, 663–671 (2006). 16680143 10.1038/ni1340

[b13] Onai N. . A clonogenic progenitor with prominent plasmacytoid dendritic cell developmental potential. Immunity 38, 943–957 (2013). 23623382 10.1016/j.immuni.2013.04.006

[b14] Cisse B. . Transcription factor E2-2 is an essential and specific regulator of plasmacytoid dendritic cell development. Cell 135, 37–48 (2008). 18854153 10.1016/j.cell.2008.09.016 PMC2631034

[b15] Ghosh H. S., Cisse B., Bunin A., Lewis K. L. & Reizis B. Continuous expression of the transcription factor e2-2 maintains the cell fate of mature plasmacytoid dendritic cells. Immunity 33, 905–916 (2010). 21145760 10.1016/j.immuni.2010.11.023 PMC3010277

[b16] Scott C. L. . The transcription factor Zeb2 regulates development of conventional and plasmacytoid DCs by repressing Id2. J Exp Med 213, 897–911 (2016). 27185854 10.1084/jem.20151715 PMC4886362

[b17] Ghosh H. S. . ETO family protein Mtg16 regulates the balance of dendritic cell subsets by repressing Id2. J Exp Med 211, 1623–1635 (2014). 24980046 10.1084/jem.20132121 PMC4113936

[b18] Bjorck P., Leong H. X. & Engleman E. G. Plasmacytoid dendritic cell dichotomy: identification of IFN-alpha producing cells as a phenotypically and functionally distinct subset. J Immunol 186, 1477–1485 (2011). 21172865 10.4049/jimmunol.1000454 PMC3138736

[b19] Kamogawa-Schifter Y. . Ly49Q defines 2 pDC subsets in mice. Blood 105, 2787–2792 (2005). 15598811 10.1182/blood-2004-09-3388

[b20] Niederquell M. . Sca-1 expression defines developmental stages of mouse pDCs that show functional heterogeneity in the endosomal but not lysosomal TLR9 response. Eur J Immunol 43, 2993–3005 (2013). 23922217 10.1002/eji.201343498

[b21] O’Keeffe M. . Mouse plasmacytoid cells: long-lived cells, heterogeneous in surface phenotype and function, that differentiate into CD8(+) dendritic cells only after microbial stimulus. J Exp Med 196, 1307–1319 (2002). 12438422 10.1084/jem.20021031 PMC2193989

[b22] Schlitzer A. . Identification of CCR9- murine plasmacytoid DC precursors with plasticity to differentiate into conventional DCs. Blood 117, 6562–6570 (2011). 21508410 10.1182/blood-2010-12-326678

[b23] Bryant C. . A CD2 high-expressing stress-resistant human plasmacytoid dendritic-cell subset. Immunol Cell Biol (2016). 10.1038/icb.2015.116 26791160

[b24] Du Q. . Preferential depletion of CD2(low) plasmacytoid dendritic cells in HIV-infected subjects. Cell Mol Immunol 8, 441–444 (2011). 21516119 10.1038/cmi.2011.9 PMC4012886

[b25] Matsui T. . CD2 distinguishes two subsets of human plasmacytoid dendritic cells with distinct phenotype and functions. J Immunol 182, 6815–6823 (2009). 19454677 10.4049/jimmunol.0802008 PMC2749454

[b26] Zhang X. . Neonatal plasmacytoid dendritic cells (pDCs) display subset variation but can elicit potent anti-viral innate responses. PLoS One 8, e52003 (2013). 23326320 10.1371/journal.pone.0052003 PMC3542339

[b27] Wilhelm T. R. . Siglec-1-positive plasmacytoid dendritic cells (pDCs) in human peripheral blood: A semi-mature and myeloid-like subset imbalanced during protective and autoimmune responses. Clin Immunol 163, 42–51 (2016). 26674280 10.1016/j.clim.2015.12.001

[b28] Schlitzer A. . Tissue-specific differentiation of a circulating CCR9- pDC-like common dendritic cell precursor. Blood 119, 6063–6071 (2012). 22547585 10.1182/blood-2012-03-418400

[b29] Loschko J. . Antigen delivery to plasmacytoid dendritic cells via BST2 induces protective T cell-mediated immunity. J Immunol 186, 6718–6725 (2011). 21555533 10.4049/jimmunol.1004029

[b30] Satpathy A. T. . Zbtb46 expression distinguishes classical dendritic cells and their committed progenitors from other immune lineages. J Exp Med 209, 1135–1152 (2012). 22615127 10.1084/jem.20120030 PMC3371733

[b31] Schlitzer A. . Identification of cDC1- and cDC2-committed DC progenitors reveals early lineage priming at the common DC progenitor stage in the bone marrow. Nat Immunol 16, 718–728 (2015). 26054720 10.1038/ni.3200

[b32] Rieger M. A., Hoppe P. S., Smejkal B. M., Eitelhuber A. C. & Schroeder T. Hematopoietic cytokines can instruct lineage choice. Science 325, 217–218 (2009). 19590005 10.1126/science.1171461

[b33] Eilken H. . Continuous long-term detection of live cell surface markers by ‘in culture’ antibody staining. Protocol Exchange doi: 10.1038/protex.2011.205 (2011).

[b34] Costa M. R. . Continuous live imaging of adult neural stem cell division and lineage progression *in vitro*. Development 138, 1057–1068 (2011). 21343361 10.1242/dev.061663

[b35] Filipczyk A. . Network plasticity of pluripotency transcription factors in embryonic stem cells. Nat Cell Biol 17, 1235–1246 (2015). 26389663 10.1038/ncb3237

[b36] Kueh H. Y., Champhekar A., Nutt S. L., Elowitz M. B. & Rothenberg E. V. Positive feedback between PU.1 and the cell cycle controls myeloid differentiation. Science 341, 670–673 (2013). 23868921 10.1126/science.1240831 PMC3913367

[b37] Nakles R. E. . Time-lapse imaging of primary preneoplastic mammary epithelial cells derived from genetically engineered mouse models of breast cancer. J Vis Exp (2013). 10.3791/50198 PMC3601039 23425702

[b38] Eilken H. M., Nishikawa S. & Schroeder T. Continuous single-cell imaging of blood generation from haemogenic endothelium. Nature 457, 896–900 (2009). 19212410 10.1038/nature07760

[b39] Failmezger H., Frohlich H. & Tresch A. Unsupervised automated high throughput phenotyping of RNAi time-lapse movies. BMC Bioinformatics 14, 292 (2013). 24090185 10.1186/1471-2105-14-292 PMC3851277

[b40] Held M. . CellCognition: time-resolved phenotype annotation in high-throughput live cell imaging. Nat Methods 7, 747–754 (2010). 20693996 10.1038/nmeth.1486

[b41] Brown K. N., Trichel A. & Barratt-Boyes S. M. Parallel loss of myeloid and plasmacytoid dendritic cells from blood and lymphoid tissue in simian AIDS. J Immunol 178, 6958–6967 (2007). 17513745 10.4049/jimmunol.178.11.6958

[b42] Brown K. N., Wijewardana V., Liu X. & Barratt-Boyes S. M. Rapid influx and death of plasmacytoid dendritic cells in lymph nodes mediate depletion in acute simian immunodeficiency virus infection. PLoS Pathog 5, e1000413 (2009). 19424421 10.1371/journal.ppat.1000413 PMC2671605

[b43] Chopin M. . RUNX2 Mediates Plasmacytoid Dendritic Cell Egress from the Bone Marrow and Controls Viral Immunity. Cell Rep 15, 866–878 (2016). 10.1016/j.celrep.2016.03.066 27149837

[b44] Sawai C. M. . Transcription factor Runx2 controls the development and migration of plasmacytoid dendritic cells. J Exp Med 210, 2151–2159 (2013). 24101375 10.1084/jem.20130443 PMC3804932

[b45] Liu K. & Nussenzweig M. C. Origin and development of dendritic cells. Immunol Rev 234, 45–54 (2010). 20193011 10.1111/j.0105-2896.2009.00879.x

[b46] Naik S. H. . Diverse and heritable lineage imprinting of early haematopoietic progenitors. Nature 496, 229–232 (2013). 23552896 10.1038/nature12013

[b47] Paul F. . Transcriptional Heterogeneity and Lineage Commitment in Myeloid Progenitors. Cell 163, 1663–1677 (2015). 26627738 10.1016/j.cell.2015.11.013

[b48] Perie L., Duffy K. R., Kok L., de Boer R. J. & Schumacher T. N. The Branching Point in Erythro-Myeloid Differentiation. Cell 163, 1655–1662 (2015). 26687356 10.1016/j.cell.2015.11.059

[b49] Oostendorp R. A. . Stromal cell lines from mouse aorta-gonads-mesonephros subregions are potent supporters of hematopoietic stem cell activity. Blood 99, 1183–1189 (2002). 11830464 10.1182/blood.v99.4.1183

[b50] Hilsenbeck O. . Software tools for single-cell tracking and quantification of cellular and molecular properties. Nat Biotechnol 34, 703–706 (2016). 27404877 10.1038/nbt.3626

