# Supplementary material for: Continuous single cell imaging reveals sequential steps of plasmacytoid dendritic cell development from common dendritic cell progenitors
Source: Sci Rep. 2016 Nov 28;6:37462. doi: 10.1038/srep37462 (PMC5124969; doi:10.1038/srep37462)
Supplement: Supplementary Information [file srep37462-s1.pdf]

Continuous single cell imaging reveals sequential steps of plasmacytoid dendritic cell development from common dendritic cell progenitors

Short title: pDC differentiation

Ezgi Dursun[a], Max Endele[b], Andrea Musumeci[a], Henrik Failmezger[c,d], Shu-Hung Wang[a], Achim Tresch[c,d], Timm Schroeder[b], Anne B Krug[a]

[a]Institute for Immunology, Biomedical Center, Ludwig-Maximilians-University Munich, Großhaderner Str. 9, 82152 Martinsried, Germany

[b]Department of Biosystems Science and Engineering, ETH Zurich, Mattenstrasse 26, 4058 Basel, Switzerland

[c]Max-Planck-Institute for Plant Breeding Research, Carl-von-Linné-Weg 10, 50829 Cologne, Germany

[d]Department of Biology, University of Cologne, Zülpicher Str. 47, 50829 Cologne, Germany

Correspondence: Anne Krug, M.D.

Institute for Immunology, Biomedical Center Munich, Ludwig-Maximilians-University, Großhaderner Str. 9, D-82152 Planegg-Martiensried, Germany

Phone: +49-89-2180-75646, E-mail: anne.krug@med.uni-muenchen.de

Supplementary Figure Legends

Supplementary Fig. 1 Sorting of CDPs from murine BM cells

(A) BM cells were isolated from 6-8 weeks old C57BL/6 mice and stained with fluorescently labelled antibodies; Lin-FITC, CD135-PE, CD115-APC, CD117-e780, CD11c-PE-Cy7 and MHCII-e450. CDPs were gated as Lin^- $CD135^+$ $CD115^+$ $CD117^{low}$ $CD11c^-$ $MHCII^-$. Gating strategy for sorting CDPs is shown. (B) Purity control was done by FACS analysis after cell sorting.

Supplementary Fig. 2 Generation of DC subpopulations in CDP cultures and monitoring of marker expression by in culture staining

(A) Phase contrast images of CDPs and EL08 stromal feeder cells in coculture system are shown. Black arrow indicates CDPs, white arrow indicates EL08 stromal cells. (B) CDPs ($CD45.2^+$) were cocultured either with total BM cells ($CD45.1^+$) or on EL08 stromal cells in the presence of 20ng/ml Flt3L for 3 days. The percentages of pDCs, cDCs and $CCR9^{high}$ pDCs and $SiglecH^+$ $CCR9^{low}$ precursors were determined by FACS analysis. Results of one representative of 3 experiments are shown. (C) Total BM cells were isolated from C57BL/6 mice and cultured in the presence of SiglecH-A647 antibody for 5 days. After 5 days, cells were stained freshly with fluorescently labelled antibodies against SiglecH, CCR9, B220, CD11c and MHCII. The percentages of $CCR9^{high}$ pDCs and $CCR9^{low}$ cells within $B220^+$ $CD11c^+$ $MHCII^{low}$ cells were determined by FACS analysis. The detection of SiglecH by in culture staining with anti-SiglecH-A647 and by additional fresh staining with anti-SiglecH-488 is shown in comparison to the FMO (fluorescence minus one) control without additional anti-SiglecH-A488 staining. Results of one representative of 2 experiments are shown.

Supplementary Fig. 3 Annotation of fluorescent marker detection in pedigrees

A representative pedigree of a single CDP and its progeny is shown. CDPs were cultured on EL08 stromal cells for 5 days, fluorescently labelled antibodies against SiglecH, CCR9, CD11c and MHCII were added at the start of the experiment time. CDP and their progeny were monitored by time-lapse microscopy and single cell tracking. All relevant information was recorded and cell types were annotated in colored lines. Phase contrast and fluorescent images of indicated cells are shown.

Supplementary Fig. 4 Sequential marker acquisition and heterogeneous division kinetic in CDP progeny

Results shown in in the main text were confirmed in a second continuous single cell imaging experiment. CDPs were cultured with Flt3L on EL08 stromal cells for 5 days and their progeny were observed continuously by single cell imaging and tracking. In culture staining for CD11c, CCR9 and Siglec H was performed and marker detection (on, off or high) was annotated in pedigrees. (A) The time points of first occurrence of fluorescent markers in all pedigrees were determined (n=45 pedigrees). (B) The time points of upregulation of fluorescent markers in all pedigrees were determined (n=45 pedigrees). Data are shown as box plots with whiskers and outliers, median values are indicated by horizontal lines (* $p<0.05$, Kruskall-Wallis test with Donn's post test). (C) Pedigrees were segregated in to CCR9^{high} pDC fate and SiglecH^{+} CCR9^{low} precursor fate. The numbers of generations in each pedigree are shown as symbols The median numbers of generations are indicated by lines. (D) The numbers of pedigrees with indicated number of generations are shown for pedigrees with CCR9^{high} pDC fate and only SiglecH^{+} CCR9^{low} precursor fate. (E) The pie chart shows the percentage of pedigrees, in which generation of pDCs or cDCs occurred,

in which CCR9^{low} SiglecH^{+} precursor cells were generated without occurrence of pDCs or cDCs, and, in which only cells with CDP or pre-DC phenotype were generated (undifferentiated).

Supplementary Fig. 5 Phenotype of CCR9^{low} SiglecH^{+} precursors and CCR9^{high} pDCs after culture with stromal cells and growth factors

SiglecH^{+} BST2^{+} CD11c^{+} B220^{+} primary bone marrow cells were seperated into CCR9^{low} precursors and CCR9^{high} pDCs by sorting to high purity (A) and each population was cultured with EL08 stromal cells and Flt3L (20 ng/ml) or Flt3L and GM-CSF (10 % supernatant of GM-CSF producing cell line corresponding to 20 ng/ml) for 48 hours. Expression of CCR9, Siglec H and B220 (gated on life CD45.2^{+} CD11c^{+} cells) and the expression of CD11b versus MHC class II was determined by flow cytometry (B). Overlay dot plots indicate CD11b versus MHCII expression of CCR9^{low} (black) CCR9^{high} cells (red) generated from CCR9^{low} SiglecH^{+} precursors after 48 hrs of culture.

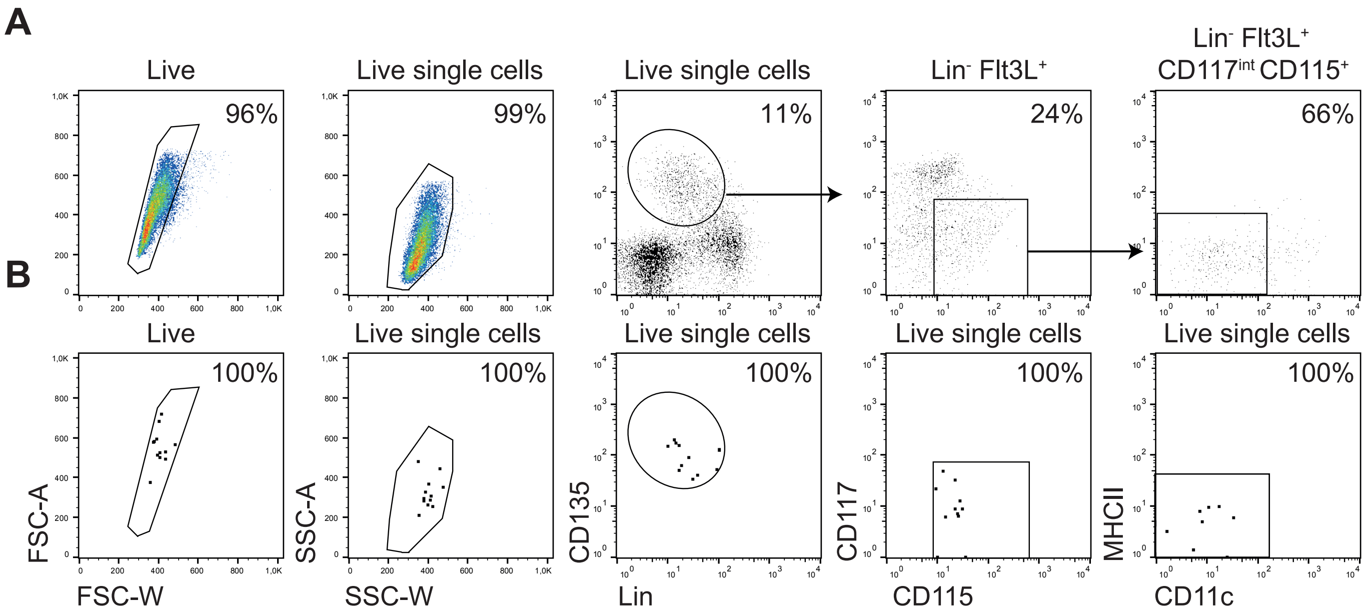

A
B
Live
Live single cells
Live single cells
Lin- Flt3L+
Lin- Flt3L+ CD117int CD115+
96%
99%
11%
24%
66%
100%
FSC-A
FSC-W
SSC-A
SSC-W
CD135
Lin
CD117
CD115
MHCII
CD11c

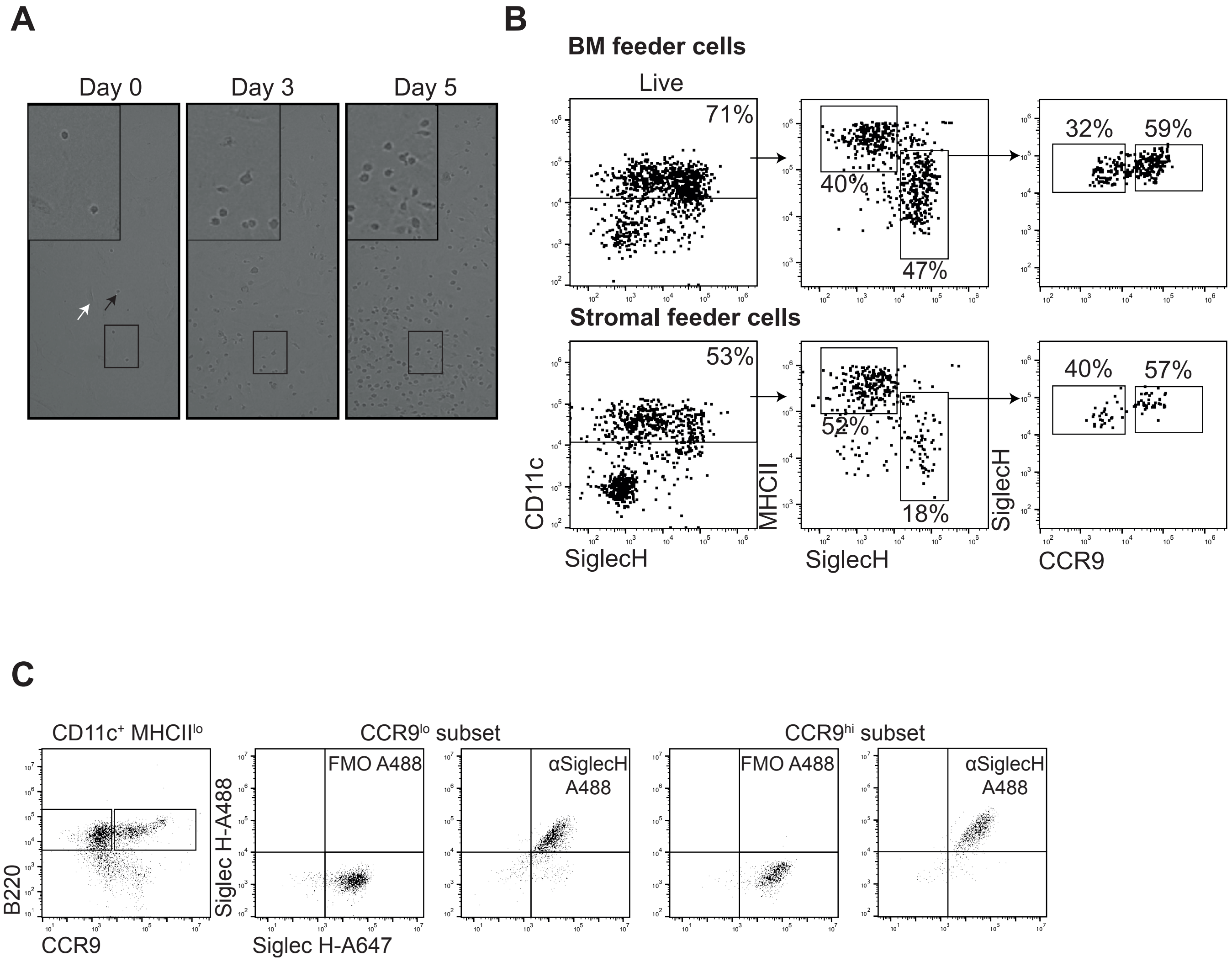
A
Day 0
Day 3
Day 5
B
BM feeder cells
Live
71%
40%
47%
32%
59%
Stromal feeder cells
53%
52%
18%
40%
57%
CD11c
SiglecH
MHCII
SiglecH
SiglecH
CCR9
C
CD11c+ MHCIIlo
CCR9lo subset
CCR9hi subset
FMO A488
αSiglecH A488
FMO A488
αSiglecH A488
B220
CCR9
Siglec H-A488
Siglec H-A647

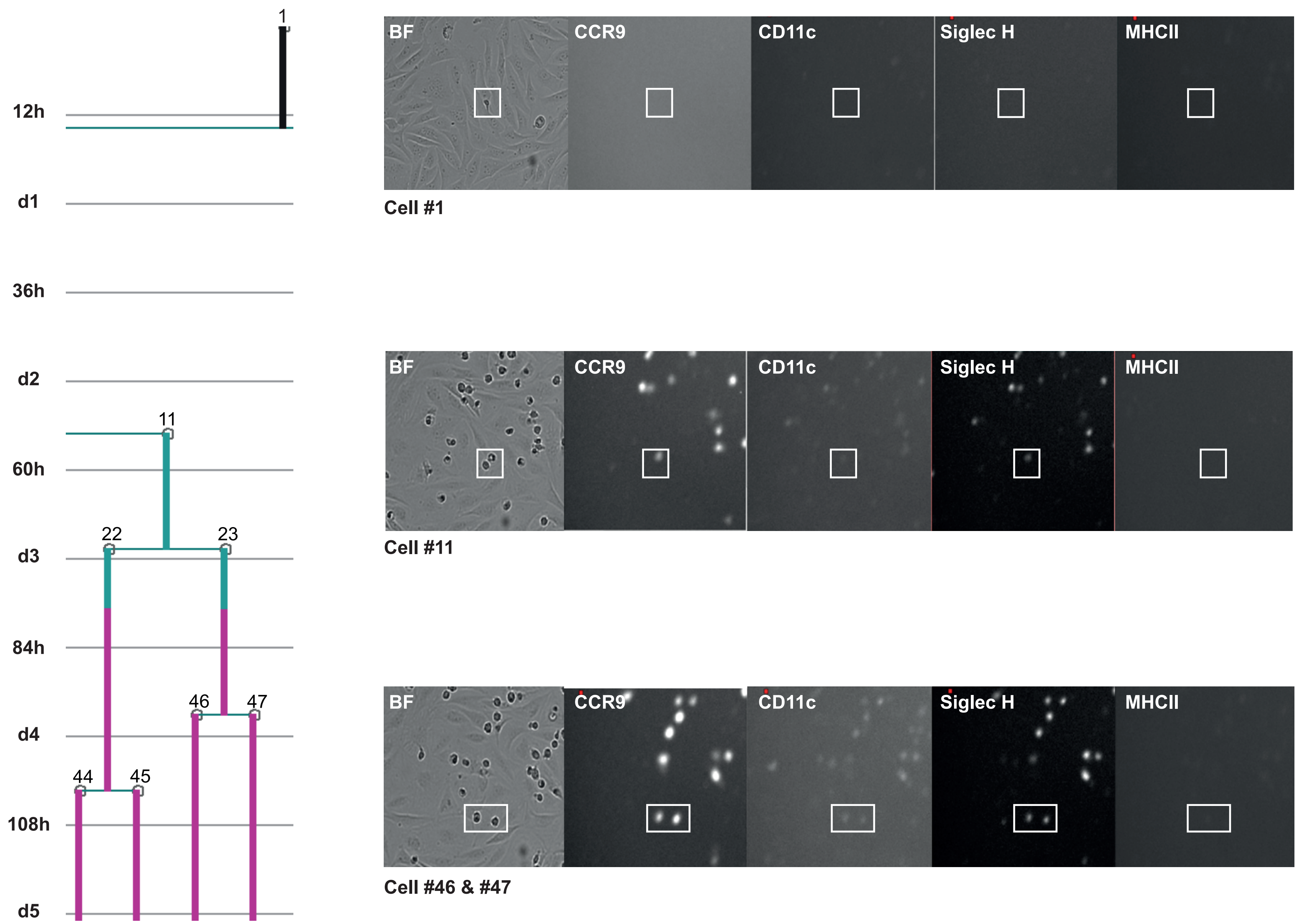
1
12h
d1
36h
d2
11
60h
22
23
d3
84h
46
47
d4
44
45
108h
d5
BF
CCR9
CD11c
Siglec H
MHCII
Cell #1
BF
CCR9
CD11c
Siglec H
MHCII
Cell #11
BF
CCR9
CD11c
Siglec H
MHCII
Cell #46 & #47

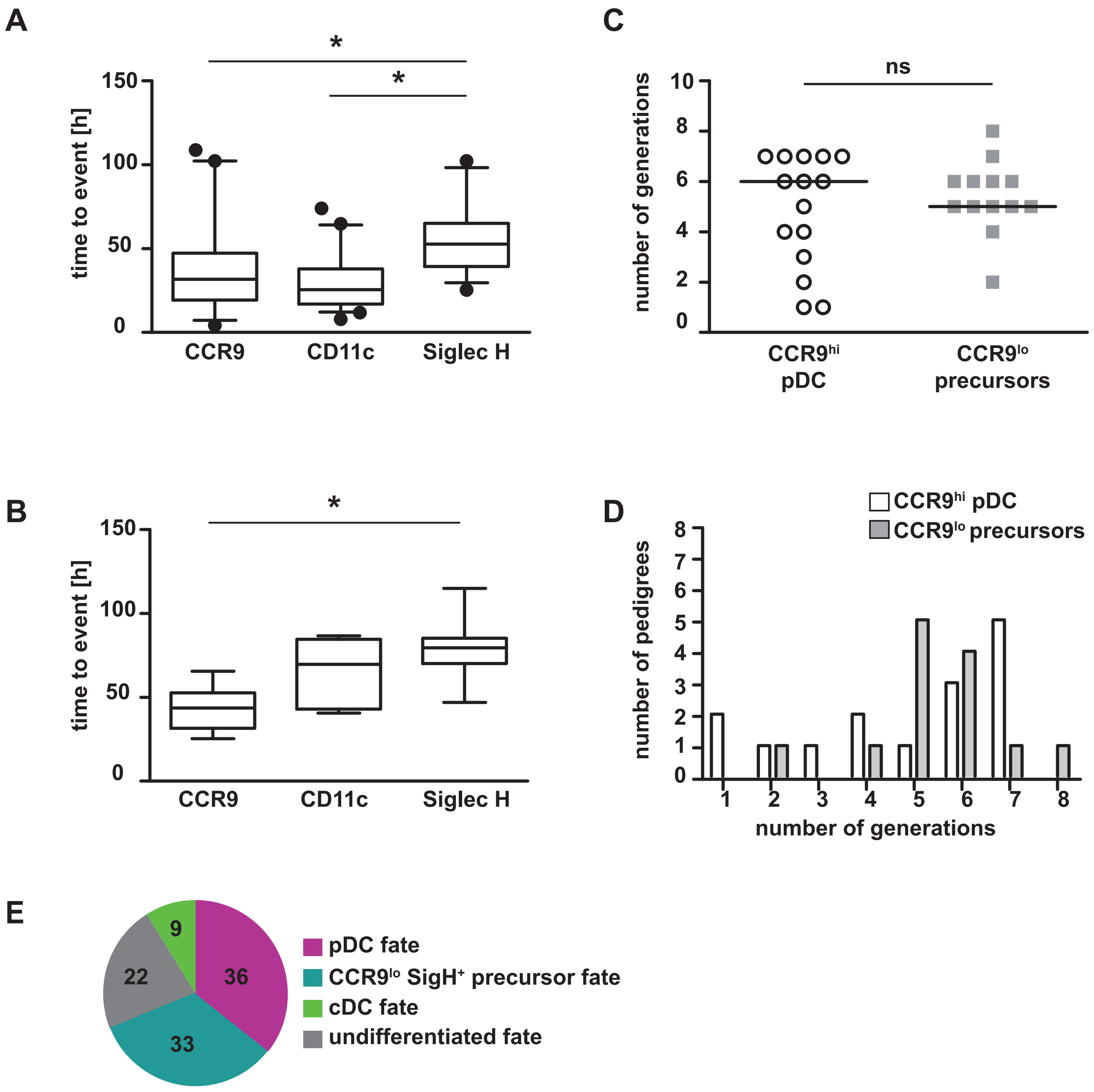
A
*
*
150
100
50
0
time to event [h]
CCR9
CD11c
Siglec H
C
ns
10
8
6
4
2
0
number of generations
CCR9hi pDC
CCR9lo precursors
B
*
150
100
50
0
time to event [h]
CCR9
CD11c
Siglec H
D
CCR9hi pDC
CCR9lo precursors
8
7
6
5
4
3
2
1
0
number of pedigrees
1
2
3
4
5
6
7
8
number of generations
E
9
22
36
33
pDC fate
CCR9lo SigH+ precursor fate
cDC fate
undifferentiated fate

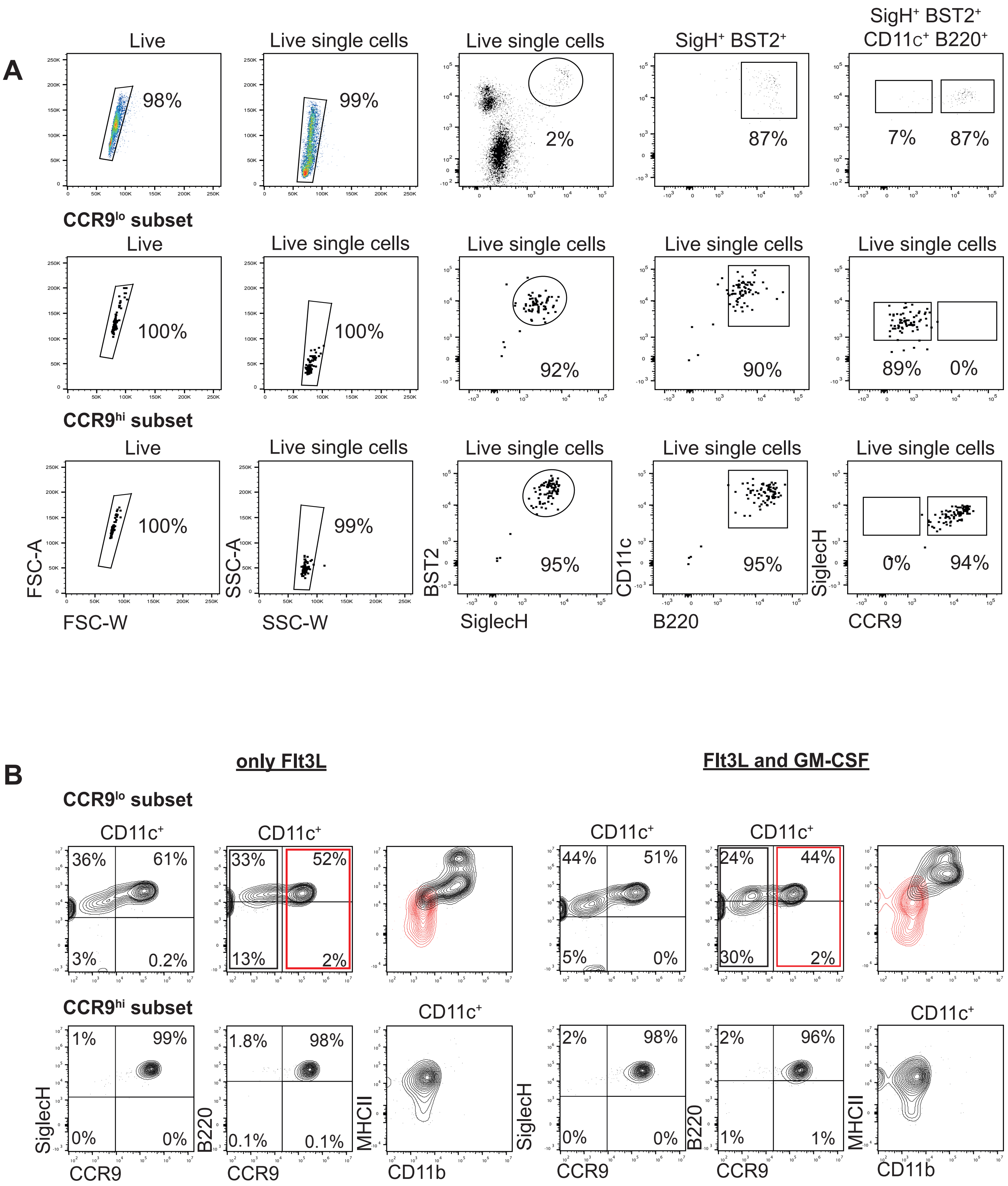
A
Live
Live single cells
Live single cells
SigH+ BST2+
SigH+ BST2+ CD11c+ B220+
98%
99%
2%
87%
7%
87%
CCR9lo subset
Live
Live single cells
Live single cells
Live single cells
Live single cells
100%
100%
92%
90%
89%
0%
CCR9hi subset
Live
Live single cells
Live single cells
Live single cells
Live single cells
100%
99%
95%
95%
0%
94%
FSC-A
FSC-W
SSC-A
SSC-W
BST2
SiglecH
CD11c
B220
SiglecH
CCR9
B
only Flt3L
Flt3L and GM-CSF
CCR9lo subset
CD11c+
36%
61%
3%
0.2%
CD11c+
33%
52%
13%
2%
CD11c+
44%
51%
5%
0%
CD11c+
24%
44%
30%
2%
CCR9hi subset
1%
99%
0%
0%
1.8%
98%
0.1%
0.1%
CD11c+
2%
98%
0%
0%
2%
96%
1%
1%
CD11c+
SiglecH
CCR9
B220
CCR9
MHCII
CD11b
SiglecH
CCR9
B220
CCR9
MHCII
CD11b

Supplementary Table 1. Transition frequency between cell types[a]

	CDP	pre-DC	CCR9^{lo} SiglecH^{+} precursor	CCR9^{lo} SiglecH^{hi} precursor	pDC	(pre)-cDC
CDP	-	54	13	0	1	3
pre-DC	2	-	47	0	2	19
CCR9^{lo} SiglecH^{+} precursor	1	1	-	17	36	2
CCR9^{lo} SiglecH^{hi} precursor	0	0	4	-	3	0
pDC	0	0	22	3	-	0
(pre)-cDC	0	0	21	0	3	-

[a]The number of transitions between cell types indicated in the left column and cell types indicated in the upper row are shown (results from exp.1)
